# Stigmatizing Beliefs and Attitudes to Depression in Adolescent School Students in Chile and Colombia

**DOI:** 10.3389/fpsyg.2020.577177

**Published:** 2020-10-23

**Authors:** Vania Martínez, Marcelo A. Crockett, Álvaro Jiménez-Molina, H. Daniel Espinosa-Duque, Elisa Barrientos, Jorge L. Ordóñez-Carrasco

**Affiliations:** ^1^Centro de Medicina Reproductiva y Desarrollo Integral del Adolescente (CEMERA), Facultad de Medicina, Universidad de Chile, Santiago, Chile; ^2^ANID, Millennium Science Initiative Program, Millennium Nucleus to Improve the Mental Health of Adolescents and Youths (Imhay), Santiago, Chile; ^3^ANID, Millennium Science Initiative Program, Millennium Institute for Research in Depression and Personality (MIDAP), Santiago, Chile; ^4^Doctorado en Salud Pública, Escuela de Salud Pública, Universidad de Chile, Santiago, Chile; ^5^ANID, Millennium Science Initiative Program, Millennium Nucleus in Social Development (DESOC), Santiago, Chile; ^6^Facultad de Psicología, Universidad Diego Portales, Santiago, Chile; ^7^Facultad de Psicología, Universidad CES, Medellín, Colombia; ^8^Departamento de Psiquiatría y Salud Mental Oriente, Facultad de Medicina, Universidad de Chile, Santiago, Chile; ^9^Departamento de Psicología, Universidad de Almería, Almería, Spain

**Keywords:** stigma, depression, adolescents, Latin America, gender differences, depression stigma scale

## Abstract

Major depressive disorder (MDD) affects between 4 and 5% of adolescents. However, there is still a huge gap between adolescents who meet criteria for MDD and those who receive mental health care. Stigmatizing attitudes toward depression are among the main barriers to seeking professional help. The aim of this article is to examine the individual characteristics associated with stigmatizing attitudes toward depression in a sample of adolescent school students from Chile and Colombia, and present the adaptation and psychometric properties of the Personal Depression Stigma Scale (DSS-Personal) for both countries. A total of 2971 adolescents, aged 10–19 (*M* = 14.6, *SD* = 1.5), who were recruited from eight schools in Santiago, Chile (*n* = 2022), and eight schools in Medellín, Colombia (*n* = 949), completed the DSS-Personal, the Patient Health Questionnaire (PHQ-9), and a questionnaire of individual sociodemographic characteristics. Factor structure, internal consistency, and validity of the DSS-Personal were assessed. Multiple linear regression models were used to evaluate the association between DSS-Personal scores and sociodemographic information, depression scores, and the use of health services by country. Confirmatory factor analysis supported the unidimensional structure of the DSS-Personal, while the estimated reliability of its scores was acceptable. Results show that DSS-Personal scores were higher in adolescents in Colombia than in Chile (*U* = 9.36, *p* < 0.001). Immigrant status was the only variable significantly related to personal depression stigma in both samples. Being female was associated with lower levels of stigma in adolescents in Chile, while depressive symptoms were associated with lower levels of stigma in adolescents in Colombia. Age, having been diagnosed with depression, and being in pharmacological or psychological treatment were not related to levels of personal depression stigma in either sample. The identified associated factors of personal depression stigma should be considered in the development of anti-stigma campaigns; also, gender differences require special attention. The results of this study suggest that it is important to offer school-based programs to reduce personal stigma, and that specific anti-stigma campaigns should address personal stigma in men and immigrants.

## Introduction

Major depressive disorder (MDD) affects between 4 and 5% of adolescents (Thapar et al., 2012) and it is estimated that up to 25% will have experienced at least one depressive episode before reaching adulthood (Kessler et al., 2001). In an epidemiologic study conducted in Santiago, Chile, the annual prevalence of depression associated with social disability for adolescents aged 12–18 was 7.8% (Vicente et al., 2012). In Medellín, Colombia, a study showed a prevalence of adolescent depression of 13.1% (CES and Alcaldía Medellín, 2009). Depression is associated with negative consequences such as functional impairment, poor school performance, difficulty in interpersonal relationships, suicidal thoughts and behaviors, physical health problems, other psychiatric disorders, and worse quality of life in adulthood (Fergusson et al., 2007; Thapar et al., 2012; McLeod et al., 2016).

Adolescence is considered a critical time for the early detection and adequate treatment of depression (Kieling et al., 2019). There are psychotherapeutic and pharmacological treatments with proven efficacy for depression in this period of life (Thapar et al., 2012; Weersing et al., 2017). Unfortunately, there is evidence for low rates of care-seeking behaviors among adolescents (Rickwood et al., 2007). Stigma and self-stigmatizing attitudes to mental illness are among the most prominent barriers to help-seeking for mental health problems (Barney et al., 2006; Clement et al., 2015), especially in adolescence (Gulliver et al., 2010; Kaushik et al., 2016). Self-stigmatization makes it difficult for adolescents to report emotional or behavioral manifestations of mental health problems in a timely manner and leads to an avoidance of interventions, resulting in poorer long-term outcomes (Kaushik et al., 2016).

Stigma has been described as a set of negative attitudes and beliefs that motivate people to fear, reject, discriminate against, and socially exclude people with mental illness (Goffman, 1963; Brohan et al., 2010). Stigma can manifest itself in various ways. People with depression may experience perceived stigma, which reflects peoples’ beliefs about the negative attitudes of others toward depression, personal stigma, which refers to the own negative feelings and attitudes toward people with depression, and self-stigma, which occurs when the stigmatized individual internalizes the negative ideas and responses of others, leading to negative thoughts and emotional reactions to themselves (Griffiths et al., 2008; Livingston and Boyd, 2010; Corrigan et al., 2012).

Multiple factors may be associated with stigma toward depression. There is evidence that stigma is greater among men, people with less education, and those with higher levels of depressive symptoms (Pyne et al., 2004; Crisp et al., 2005; Griffiths et al., 2008). Likewise, it has been suggested that personal stigma toward physical and mental health issues is more prevalent in immigrant populations (Griffiths et al., 2008; Henderson, 2016). Personal stigma may be associated with higher levels of depressive symptoms, greater psychological distress, and poor quality of life (Mak et al., 2007; Griffiths et al., 2008; Livingston and Boyd, 2010; Boyd et al., 2014; Lien et al., 2015).

Although stigma in mental health remains a global problem, there is evidence that the sociocultural environment (collective and individual values, ideals, norms, and social expectations) may shape the way stigma is expressed in different social groups and modulate its severity (Yang et al., 2007, 2013; Lien et al., 2015; Chang et al., 2016; Mascayano et al., 2020). Therefore, it is important to conduct studies with populations in a variety of sociocultural contexts. While most stigma-related research has been conducted in Europe and North America, over the last decade there has been a significant increase in information about the stigma associated with mental disorders in Latin America (Mascayano et al., 2016). These studies have shown that Latin America and developing countries are characterized by high levels of public and self-stigmatization toward mental illness (Alonso et al., 2008; Mascayano et al., 2016).

In Chile and Colombia some studies have shown a high presence of stigmatizing beliefs and attitudes toward mental disorders, which have been associated with a reduction in seeking help and accessing to health services (Uribe Restrepo et al., 2007; Álvarez Ramírez and Almeida Salinas, 2008; Yang et al., 2013; Mascayano et al., 2016; Hernández Holguín and Sanmartín Rueda, 2018; Sapag et al., 2018; Campo-Arias et al., 2020). In Chile, one of the specific objectives of the 2017–2025 National Mental Health Plan is “to reduce the stigma associated with mental health problems” (MINSAL, 2017, p. 75), which includes initiatives such as an evaluation of mental health-related stigma in primary health care. A national survey by the Colombian Ministry of Health shows that about 50% of the population reports that personal stigma along with limited availability of services is one of the main barriers to accessing mental health services (MINSALUD, 2015). In this context, stigma has been recognized by policy makers and organizations in Chile and Colombia as an important public health issue (MINSALUD, 2015; MINSAL, 2017); also, some guidelines and psychosocial interventions have been developed for reducing stigma in mental health care (Yang et al., 2013; Rodríguez Araújo, 2014; Schilling et al., 2015; Sapag et al., 2018).

Despite these advances in research in the Latin American context, there is still a lack of research on stigmatizing beliefs and attitudes toward depression in adolescents. This lack of knowledge makes it difficult to design anti-stigma campaigns targeting this population and implement effective interventions to improve the management of adolescent depression.

Likewise, despite the increase in research on stigma associated with mental illness in developed countries, methodological discrepancies between existing studies constitute a major limitation (Kaushik et al., 2016), especially because multiple instruments have been used to measure personal stigma (Watson et al., 2007; Rüsch et al., 2010; Corrigan and Rao, 2012).

The Depression Stigma Scale (DSS; Griffiths et al., 2004) is a brief questionnaire commonly used to assess depression stigma in the general population and people with depression. Since the DSS is already used in other countries (Griffiths et al., 2006, 2008; Dietrich et al., 2014; Boerema et al., 2016), this scale allows cross-cultural comparisons. Currently, there are few studies in adolescents (e.g., Calear et al., 2011; Dardas et al., 2017; Howard et al., 2018) and, furthermore, no research has been conducted in Latin America using the DSS.

The DSS comprises a 9-item Personal Stigma subscale (DSS-Personal) that assesses people’s personal beliefs and attitudes toward depression and a 9-item Perceived Stigma subscale that assesses people’s beliefs about others’ attitudes toward depression. Previous research highlights the importance of measuring and validating the concepts of personal and perceived stigma separately, while also estimating predictors and designing interventions independently for each dimension of stigma (Griffiths et al., 2008; Yap et al., 2014; Boerema et al., 2016).

In this context, the aim of this study is to examine the individual characteristics associated with stigmatizing attitudes toward depression in a sample of adolescent school students from Chile and Colombia, and present an adaptation of the DSS-Personal for both countries along with its psychometric properties.

Since this study was conducted with adolescent population in a school setting, and not with a clinical population treated in health centers, we decided to explore the factors associated with personal stigma, a dimension that is more likely to be modified by school-based interventions, including Internet-based programs (Corrigan et al., 2012; Griffiths et al., 2014). Additionally, the literature on perceived stigma is more inconsistent than that on personal stigma, especially due to sociodemographic factors (Griffiths et al., 2008); furthermore, prior research has shown that the personal stigma construct works relatively well in multiple populations and cultural contexts (Griffiths et al., 2006; Dietrich et al., 2014; Boerema et al., 2016).

## Materials and Methods

### Participants and Setting

The data were collected as part of the baseline assessment of two randomized controlled trials (one in Chile and one in Colombia) to evaluate the efficacy of “*Cuida tu Ánimo*” (“Take Care of Your Mood,” in English), an Internet-based program for prevention and early intervention of adolescent depression (Parada et al., 2020). The inclusion criteria for the schools were: be coeducational, have at least two classes per course, have no more than 60% students of one sex, and have a counselor or psychologist. In Santiago, Chile, State-subsidized schools from municipalities in the north of the city were invited to participate. Eight out of 20 invited schools met the inclusion criteria and agreed to participate. In Medellín, Colombia, public schools were invited to participate in collaboration with the Ministry of Education. Eight out of 12 invited schools met the inclusion criteria and agreed to participate. All students within the same class were invited to participate in the study. The participants were 2971 adolescents, from 6th to 11th grades, 2022 from Santiago, Chile, and 949 from Medellín, Colombia. A total of 207 classes participated in the study, 85 in Chile, and 122 in Colombia. Overall, 52.2% of the participants were female, their mean age was 14.6 years (±1.5), and 6.5% were immigrants (Table 1). All participants spoke Spanish.

**TABLE 1:** Characteristics of the sample.

	**Chile *n* = 2022**	**Colombia *n* = 949**	**Total *n* = 2971**
**Sex (%)**			
Male	50.5	42.3	47.8
Female	49.5	57.7	52.2
Age [mean (SD)]	15.2 (1.0)	13.4 (1.7)	14.6 (1.5)
**Lives with (%)**			
Both parents	55.8	43.3	51.9
Mother or Father	40.0	51.2	43.5
Other	4.2	5.5	4.6
Immigrant status (%)	6.6	6.3	6.5
History of depression (%)	16.5	17.1	16.7
Current psychological treatment (%)	9.7	10.4	9.9
Current pharmacological treatment (%)	2.7	2.9	2.7
PHQ-9 scores [mean (SD)]	9.1 (5.9)	9.0 (5.8)	9.1 (5.9)
DSS-Personal scores [mean (SD)]	11.3 (4.5)	13.2 (5.1)	11.9 (4.8)

*SD, standard deviation.*

### Measures

#### Depression Stigma Scale (DSS; Griffiths et al., 2004)

The DSS is a self-report instrument composed of two 9-item subscales, Personal and Perceived stigma, that measure one’s own and others’ attitudes to depression, respectively. The Personal subscale (DSS-Personal) was used in this study. It has a 5-item response format (from 0 = *strongly disagree* to 4 = *strongly agree*). The total score is composed of the sum of its item scores. A higher score indicates greater stigma.

The DSS was developed in Australia and has been used in several countries (e.g., Australia, Japan, Germany, Netherlands) and populations (e.g., national survey, local community, and distressed subset of a local community) (Griffiths et al., 2004, 2006, 2008; Dietrich et al., 2014; Boerema et al., 2016). The DSS-Personal subscale has shown adequate psychometric properties: 0.71 test-retest reliability, 0.76 internal consistency (Griffiths et al., 2004), and *r* = 0.53 convergent validity with a measure of social distance (Griffiths et al., 2008). In adolescent samples, DSS-Personal subscale scores have shown low (α = 0.54; Dardas et al., 2018) to moderate (α = 0.70–0.79; Calear et al., 2011; Howard et al., 2018) internal consistencies. In this study, the DSS was translated into Spanish through a multi-stage forward and backward procedure. Two independent bilingual people from Chile and Colombia translated the questions from the original English questionnaire into Spanish. Differences in translation were discussed and a consensus version was generated. This version was then translated back into English by a third bilingual person and compared with the original version of the DSS. Potential differences were again discussed by two authors (VM and HDE) in order to have only one version for Chile and Colombia.

#### Patient Health Questionnaire-9 (PHQ-9; Johnson et al., 2002)

The PHQ-9 is a self-report questionnaire composed of 9 items. It is used for the evaluation of depressive symptoms according to the Diagnostic and Statistical Manual of Mental Disorders-IV criteria. It has a 4-point ordinal scale (from 0 = *not at all* to 3 = *nearly every day*). Total scores are composed of the sum of the items, which can range from 0 to 27. Higher scores indicate greater severity of depression. In this study, the PHQ-9 had an internal consistency of α = 0.87, and Spearman-Brown coefficient = 0.89 for the sample in Chile, and α = 0.84, and Spearman-Brown coefficient = 0.87 for the sample in Colombia.

#### Mental Health Service Utilization

Three self-report questions about mental health service utilization were included: history of treatment for depression (*Have you ever received any type of depression treatment sometime in your life?*), pharmacological (*Are you currently being treated with any antidepressant medication (e.g., fluoxetine, sertraline, escitalopram, citalopram, venlafaxine, and bupropion)?*), and psychological treatment for depression (*Are you currently in treatment with a psychologist (psychotherapy) outside of school?*). They had a two-choice response format (1 = *yes*, 0 = *no*).

#### Sociodemographic Variables

A self-report questionnaire was included with the rest of the instruments. The sociodemographic variables considered were sex (0 = *male*, 1 = *female*), age (in years), living with parents (1 = *both parents*, 2 = *mother or father*, and 3 = *other*), and immigrant status of the participating adolescents, operationalized as having a nationality other than that of one’s country of residence (0 = *non-immigrant*, 1 = *immigrant*).

### Procedure

All procedures were approved by the Ethics Review Boards of both participating Universities. Ethical approval was obtained from the Ethics Committee of Human Research of the Faculty of Medicine of the Universidad de Chile (Chile) and the Institutional Ethics Committee of Human Research of the CES University (Colombia). Informed consent was obtained from young people over 18 years of age, while informed assent was obtained from minors, along with informed consent from their parents or primary caregivers. The questionnaires were answered by the adolescents on school computers, supervised by a member of the research team.

### Data Analysis

Descriptive statistics of the DSS-Personal items were estimated, along with the corrected item-test correlation. Univariate normal distribution of the items was assessed using the Kolmogorov-Smirnov test. Since univariate normal distribution was not accomplished (*p* < 0.05), multivariate normal distribution was rejected. The psychometric properties of the DSS-Personal subscale were assessed using Confirmatory Factor Analysis (CFA) for both samples separately. Since the item response format is on an ordinal Likert scale and multivariate normality was not achieved, we used the Unweighted Least Squares method of estimation. Comparative Fit Index (CFI), Tucker-Lewis Index (TLI), Normed Fit Index (NFI), Adjusted Goodness of Fit (AGFI), Root Mean Square Residual (RMR), and Root Mean Square Error of Approximation (RMSEA) were used to assess model fit. Values of CFI, TLI, and NFI > 0.95, AGFI > 0.90, RMR < 0.06, and RMSEA < 0.08 were considered an acceptable model fit (Hu and Bentler, 1999). Measurement invariance was tested across samples. A decrease in CFI equal to or greater than 0.01 indicated that the more restrictive model should be rejected (Cheung and Rensvold, 2002). Internal reliability of the subscale was estimated using Cronbach’s alpha and the Spearman-Brown coefficient. Validity evidence based on relations with other variables was explored using a correlation matrix that included all the study variables. The Spearman correlation for continuous non-normally distributed variables, the point-biserial correlation for continuous-categorical variables, and the phi coefficient for categorical variables were used.

Descriptive statistics were used to detail the sample characteristics, PHQ-9 score, and DSS-Personal stigma subscale score. To compare the characteristics of the Chilean and Colombian samples, the χ^2^ test was used for categorical variables and the Mann-Whitney *U* test for continuous variables (PHQ-9, DSS-Personal, and age), since they were not normally distributed within each sample according to the Kolmogorov-Smirnov test (*p* < 0.001). To further evaluate the differences between DSS-Personal scores by sex and country, the Mann-Whitney *U* test was also used, since they were not normally distributed (Kolmogorov-Smirnov test, *p* < 0.001). Multiple linear regression models were used to evaluate the association between DSS-Personal scores and sociodemographic information, depression scores, and the use of health services by country. The regression models were stratified by country due to differences in the distribution by sex and age in both samples (*p* < 0.001) and as a result of unmeasured cultural differences. Most of the variables had complete data, except for age, which had 0.03% of missing data. No imputation method was used. The psychometric properties of the DSS-Personal were examined using JASP (version 0.13.1) and AMOS v.24, while the rest of the analyses were performed in Stata 13.

## Results

Regarding the characteristics of the sample (Table 1), the proportion of males/females and the mean age were different by country (*p* < 0.001), with the Colombian sample having more female and younger adolescents. The proportion of adolescents living with both parents and with just one parent was different in both samples (*p* < 0.001). The proportion of immigrant status of the adolescents was similar between samples (*p* = 0.754). In total, 98.5% of the immigrants in the Chilean sample and 79% in the Colombian sample came from other Latin American countries.

The proportion of adolescents with a history of depression and in current psychological and pharmacological treatment for depression was similar in both samples (*p* < 0.785). The PHQ-9 scores were also similar in both samples (*p* = 0.444).

### Personal Depression Stigma Subscale

The descriptive statistics of the DSS-Personal items are presented in Table 2. According to the CFA, the 9-item DSS-Personal subscale had a poor fit for the Chilean sample (CFI = 0.76; TLI = 0.68; NFI = 0.76; AGFI = 0.91, RMR = 0.11; and RMSEA = 0.15), and the Colombian sample (CFI = 0.94; TLI = 0.93; NFI = 0.94; AGFI = 0.97, RMR = 0.78; and RMSEA = 0.10). Since the 9-item factor structure had a poor fit, a version of the DSS-Personal with fewer items was tested. Items 1 and 7 were dropped because their corrected item-test correlation was lower than 0.20. Item 7 in the Colombian sample had a corrected item-test correlation of 0.22, but in order to have the same set of items in both samples, it was dropped for the analysis. The one-factor solution of the 7-item subscale was satisfactory for both the Chilean sample (CFI = 0.99; TLI = 0.98; NFI = 0.99; AGFI = 0.99; RMR = 0.03; and RMSEA = 0.04), and the Colombian sample (CFI = 0.99; TLI = 0.98; NFI = 0.98; AGFI = 0.99; RMR = 0.05; and RMSEA = 0.06) after adding a covariance term between the errors of items 2 and 3 and those of items 8 and 9. Both pairs of items have semantic similarities that could explain the need to add the covariance term.

**TABLE 2:** Descriptive statistics for DSS-Personal items by sample.

	**Chile**					**Colombia**				
	**Mean**	**SD**	**Skew**	**Kurt**	**Item-test cor**	**Mean**	**SD**	**Skew**	**Kurt**	**Item-test cor**
1. People with depression could snap out of it if they wanted	2.99	1.12	–1.02	3.31	0.12	2.88	1.11	–0.83	3.02	0.19
2. Depression is a sign of personal weakness	2.31	1.29	–0.36	2.09	0.36	2.58	1.12	–0.65	2.73	0.27
3. Depression is not a real medical illness	1.96	1.23	0.05	2.13	0.26	2.20	1.23	–0.05	2.03	0.26
4. People with depression are dangerous	1.38	1.12	0.49	2.62	0.41	1.77	1.21	0.21	2.22	0.46
5. It is best to avoid people with depression so that you don’t become depressed yourself	0.97	1.11	1.01	3.28	0.42	1.48	1.29	0.53	2.21	0.55
6. People with depression are unpredictable	2.23	0.99	–0.18	2.96	0.25	2.18	1.08	–0.11	2.53	0.40
7. If I had depression I would not tell anyone	1.83	1.32	0.18	1.91	0.03	2.05	1.36	–0.03	1.80	0.22
8. I would not employ someone if I knew they had been depressed	1.15	1.12	0.79	2.93	0.37	1.36	1.26	0.67	2.43	0.47
9. I would not vote for a politician if I knew they had been depressed	1.30	1.14	0.55	2.61	0.38	1.62	1.26	0.40	2.23	0.48

*SD, standard deviation; Skew, skeweness; Kurt, Kurtosis; Item-test cor, corrected item-test correlation.*

Using the 7-item DSS-Personal subscale, measurement invariance was tested across samples, but only configural invariance was met (χ^2^ = 105.640; *df* = 24; CFI = 0.99, TLI = 0.98; and RMSEA = 0.05), indicating that the factor structure of Personal Stigma was equal for both groups, unlike the other types of measurement invariance (decrease in CFI = 0.025 for scalar invariance).

The reliability of the 7-item subscale scores was α = 0.65 and Spearman-Brown coefficient = 0.69 for the sample in Chile, and α = 0.70 and Spearman-Brown coefficient = 0.74 for the sample in Colombia.

In order to obtain validity evidence based on the relationship with other variables, correlations of the study variables are presented in Table 3. The top right section presents the correlations from the Chilean sample, while the bottom left section shows the correlations from the Colombian sample. DSS-Personal scores had weak but statistically significant correlations with other variables in both samples, except for age and current pharmacological treatment in both samples and current psychological treatment in the Colombian sample.

**TABLE 3:** Correlation matrix of study variable.

	**1**	**2**	**3**	**4**	**5**	**6**	**7**	**8**
1. DSS-Personal	–	−0.06**	–0.04	−0.14***	0.07**	−0.05*	–0.01	−0.06**
2. PHQ-9 scores	−0.16***	–	0.06**	0.30***	–0.02	0.28***	0.13***	0.20***
3. Age	–0.04	0.26***	–	–0.04	−0.07**	0.09***	0.06**	0.04
4. Female sex	−0.09**	0.20***	–0.02	–	0.05*	0.12***	–0.01	0.07**
5. Immigrant status	0.10**	–0.05	0.01	–0.01	–	−0.07**	−0.04*	−0.08***
6. History of depression	−0.07*	0.35***	0.14***	0.12***	−0.07*	–	0.30***	0.26***
7. Current pharmacological treatment	–0.04	0.19***	0.02	0.03	–0.04	0.29***	–	0.30***
8. Current psychological treatment	–0.00	0.14***	–0.00	0.03	–0.05	0.27***	0.34***	–

*The left-lower columns from the diagonal present the correlations in the Colombian sample, and the right-upper columns the correlation in the Chilean sample. *^∗^p* < 0.05; ^∗∗^*p* < 0.01; ^∗∗∗^*p* < 0.001.*

### Factors Associated With Personal Depression Stigma

The participants’ DSS-Personal scores were higher in Chile than in Colombia (*U* = 9.36, *p* < 0.001). DSS-Personal scores were higher in males than in females in the total sample ($\bar{x}$_*male*_ = 12.46 vs. $\bar{x}$_*female*_ = 11.39, *U* = 6.18, *p* < 0.001), in the Chilean sample ($\bar{x}$_*male*_ = 11.95 vs. $\bar{x}$_*female*_ = 10.64, *U* = 6.53, *p* < 0.001), and in the Colombian sample ($\bar{x}$_*male*_ = 13.74 vs. $\bar{x}$_*female*_ = 12.77, *U* = 2.93, *p* = 0.003).

Regarding the correlates of DSS-Personal scores from multiple linear regression models (Table 4), immigrant status was the only variable significantly related to personal depression stigma in both samples, meaning that immigrant adolescents had higher DSS-Personal scores than non-immigrant adolescents.

**TABLE 4:** Multiple linear regression models to evaluate the correlates of depression stigma by country.

	**Chile**				**Colombia**			
	***B***	**CI 95% *B***	**β**	***p***	***B***	**CI 95% *B***	**β**	***p***
PHQ-9 scores	−0.01	−0.04, 0.03	−0.01	0.710	−0.16	−0.22, −0.10	−0.19	<0.001
Age	−0.14	−0.34, 0.06	−0.03	0.157	−0.05	−0.24, 0.13	−0.02	0.573
Female sex	−1.28	−1.69, −0.87	−0.14	<0.001	−0.59	−1.25, 0.07	−0.06	0.077
Immigrant status	1.28	0.48, 2.07	0.07	0.002	1.93	0.63, 3.23	0.09	0.004
History of depression	−1.18	−0.76, 0.40	−0.01	0.540	0.15	−0.79, 1.10	0.01	0.749
Current pharmacological treatment	0.31	−1.00, 1.62	0.01	0.645	−0.35	−2.43, 1.73	−0.01	0.743
Current psychological treatment	−0.66	−1.38, 0.48	−0.04	0.068	0.51	−0.61, 1.63	0.03	0.368

*CI, confidence intervals.*

On the other hand, differences were also observed between the two social contexts. Depression scores were negatively related to depression stigma in adolescents in Colombia, while being female was related to lower DSS-Personal scores in adolescents in Chile. However, the *p*-value of being female bordered on a statistically significant value in the Colombian sample.

Age and service utilization variables (history of depression, current psychological and pharmacological treatment) were not associated with depression stigma in either sample. However, the *p*-value of current psychological treatment bordered on a statistically significant value in the Chilean sample.

The models were statistically significant for Chile [*F*(7,2013) = 8.93, *p* < 0.001], and Colombia [*F*(7,941) = 7.64, *p* < 0.001], but the independent variables explained only 2.7% of the variance (adjusted *R*^2^) in the Chilean sample, and 4.7% in the Colombian sample.

## Discussion

Regarding the psychometric properties of the DSS-Personal subscale, our research findings support the one-factor structure of the 7-item DSS-Personal for Colombian and Chilean adolescents, with an adequate internal consistency in the Colombian sample, but lower in the Chilean one. Our results on the one-factor structure of the DSS-Personal subscale are consistent with those obtained by the authors of the DSS (Griffiths et al., 2004).

According to our review of the literature, there are no published studies with adolescent-only samples that have examined the factor structure of the scale using CFA. In adults, a one-factor structure of the DSS-Personal subscale has been reported by a study with a Chinese community sample (Yang et al., 2020), but other studies have failed to reach a one-factor solution and have proposed that the DSS-Personal subscale could be composed of two (Zhu et al., 2019) and three factors (Boerema et al., 2016). A study with a sample comprising adolescents aged 15 and adults up to 25 years of age also yielded a two-factor solution for the DSS-Personal subscale (Yap et al., 2014). In this regard, the factor structure of the scale was examined by its authors using Principal Component Analysis (Griffiths et al., 2004, 2008), which could explain the differences in the number of factors of the DSS-Personal observed in the aforementioned studies. In addition, the reliability coefficients obtained in our study are similar to those of other studies with samples of adolescents aged 12–17 years (α = 0.54–0.70; Calear et al., 2011; Dardas et al., 2018), while better values have been found with adolescents aged 16–19 years (α = 0.79; Howard et al., 2018). These results show that it is necessary to continue exploring the psychometric properties of the DSS-personal subscale. It might be advisable to reformulate its content in order to have an instrument with proven validity, adequate internal coherence in terms of scores, and factor invariance among different cultures and age groups.

Likewise, the results show similarities and differences regarding the variables associated with personal depression stigma in samples of adolescents from two Latin American countries. First, in Chile and Colombia, the highest levels of stigma were associated with the immigrant status of adolescents. Second, being female was associated with lower levels of stigma in adolescents in Chile, while the presence of depressive symptoms was associated with lower levels of stigma in adolescents in Colombia. Likewise, age, having been diagnosed with depression, and being in pharmacological or psychological treatment were not related to levels of personal depression stigma in either sample. Interestingly, a previous study indicated that people reporting a history of depression showed lower personal stigma and that the level of current psychological distress was associated with higher personal stigma (Griffiths et al., 2008).

DSS-Personal scores were higher in Colombia than in Chile. This difference may be associated with the disparity between both countries’ levels of social development and depth of knowledge about mental health. In fact, Chile has the highest human development index and the greatest level of development of mental health services in primary care centers in Latin America (Minoletti and Sepúlveda, 2017). While some studies have reported that perceived stigma is more prevalent in countries with lower levels of socio-economic development (Alonso et al., 2008), the difference in personal stigma between both countries should be interpreted with caution and corroborated in future studies, since full measurement invariance was not held across samples in this study.

In the bivariate analysis in both samples, statistically significant gender differences were observed with respect to DSS-Personal scores, with women obtaining lower scores than men. When controlling for the other variables in the multiple regression models, these differences were statistically significant for the Chilean sample and bordered on significance in the Colombian sample. These results are consistent with those reported by Calear et al. (2011) in a sample of Australian adolescents, but not with those found by Dardas et al. (2017), who observed no gender differences in the DSS-Personal subscale in a sample of Arab adolescents. Although there is no strictly direct relationship between gender and stigma in mental health among young people, men in general tend to be more stigmatized and stigmatizing than women, which may be due to lower awareness of depression than females and socially embedded gender constructs that hold that men should handle their mental problems on their own (Kaushik et al., 2016). Likewise, phenomena such as “machismo” [macho culture] and the “culture of honor” in Latin America may be associated with gender role expressions of stigma (Yang et al., 2013), where men often have a cultural mandate to show that they are emotionally strong and hide their feelings (Mascayano et al., 2015).

Another interesting finding is that personal stigma is greater among immigrant adolescents, most of them Latin Americans. Previous studies have shown that stigma toward people with mental disorders can be present in immigrant communities, which has significant consequences for their health, exacerbating their vulnerability and health inequities (Henderson, 2016). This finding shows that stigmatization can also occur within stigmatized populations. In the study conducted by Calear et al. (2011), it was found that adolescents who did not speak English as their first language had higher levels of stigma toward depression, which could be in line with our results. The literature suggests that personal stigma increases in adolescents who perceive less control over their mental health difficulties and in families where parents have stigmatizing attitudes toward mental health problems (Kaushik et al., 2016), which could be the case for immigrant families in Chile and Colombia. Likewise, young people’s attitudes have been shown to have specific associations with those of their parents (Jorm and Wright, 2008). This interpretation requires further exploration, since during the last 5 years there has been a significant increase in migration in Chile and Colombia (Chilean Department of Foreign Affairs, 2020).

A common concern in this field is that questionnaires for measuring stigma may be especially susceptible to social desirability bias. However, research suggests that self-administered questionnaires for assessing stigma may avoid this social desirability bias (Michaels and Corrigan, 2013). In this study, the questionnaire was administered under conditions of confidentiality and anonymity, which may have made it less likely for respondents to modify their answers due to social desirability.

To our knowledge, this study is one of the first to explore personal depression stigma in adolescents in Latin American countries. However, there are some limitations that need to be considered when interpreting the results. The first one concerns the cross-sectional design of the study, which does not make it possible to establish causal relationships. Secondly, we did not explore other factors that could be associated to personal depression stigma like mental health literacy or contact with close ones who have had depression. Third, potential confounders like psychiatric illness were not assessed. Another limitation of our study derives from the low internal consistencies of the DSS-Personal subscale, especially in the Chilean sample. Likewise, the variables considered in this study explain little of the variance of the DSS-Personal subscale scores. Therefore, future studies should continue to explore other variables that may be related to the stigma of depression in adolescents. The findings of the present study suggest that additional research is needed to examine the psychometric properties and validity of the DSS-Personal subscale and its use in other Latin American samples.

Despite these limitations, our results suggest that attitudes of personal stigmatization toward depression are culturally sensitive, which should be further explored in future qualitative research. The results of this study are consistent with the notion that immigrant adolescents are especially susceptible to stigmatizing personal beliefs regarding depression (Griffiths et al., 2008). In societies with increasing ethnic diversity levels, as is the case today in Chile and Colombia, culturally homogeneous intervention strategies are likely to fail, as the attitudes of adolescents involved in the stigmatization process are influenced by cultural beliefs.

Since stigmatizing beliefs and attitudes toward depression are responsible for substantial distress and reluctance to seek appropriate help among adolescents (Gulliver et al., 2010; Kaushik et al., 2016), anti-stigma programs should not be limited to public health campaigns but should also be implemented in school settings. Educational interventions, either alone or combined with other interventions, have been consistently associated with a reduction in personal depression stigma, especially in young people (Griffiths et al., 2014). The findings of this study highlight that individual differences associated with personal stigmatizing beliefs and attitudes to depression should be considered in the development of these programs. These interventions can be more effective if they target specific groups that are most at risk of personal stigmatization, including men and immigrants (Griffiths et al., 2008, 2014). Digital technologies could be an effective complement to stigma reduction programs in school settings. In fact, Internet-based anti-stigma interventions have been shown to be as effective as those conducted by other means (Griffiths et al., 2014). This has important implications, since online interventions in mental health can be carried out more flexibly and with fewer material and human resources (Jiménez-Molina et al., 2019).

## Conclusion

Stigmatizing attitudes toward depression were found to be associated with the immigrant status of the adolescents in the Chilean and Colombian samples, while being female and having depression were associated differently across samples.

The results of this study suggest that it is important to offer school-based programs to reduce personal stigma, and that the development of psychosocial programs against stigmatizing beliefs and attitudes toward depression should be gender-sensitive and consider relevant sociocultural features of each community, especially the cultural beliefs of immigrant populations regarding mental health problems.

Additionally, using the scores of Chilean and Colombian adolescent school students, this study examined the validity and internal consistency of the Spanish-language adaptation of the 7-item DSS-Personal stigma subscale. While the results obtained support the use of the DSS-Personal stigma subscale in both countries, future studies should keep exploring the psychometric properties of the DSS, striving to improve it in order to ensure the availability of a reliable and valid instrument for assessing depression stigma in multiple cultures and age groups.

## Data Availability Statement

The raw data supporting the conclusions of this article will be made available by the authors, without undue reservation.

## Ethics Statement

The studies involving human participants were reviewed and approved by the Ethics Committee of Human Research, Faculty of Medicine, Universidad de Chile, Chile and the Institutional Ethics Committee of Human Research, CES University, Colombia. Written informed consent to participate in this study was provided by the participants, and where necessary, the participants’ legal guardian/next of kin.

## Author Contributions

VM was the principal investigator of the original research project (“Cuida tu ánimo”) in Chile and conceived this study. HDE-D was the principal investigator of the original research project (“Cuida tu ánimo”) in Colombia. MAC and JO-C performed the statistical analyses. All authors analyzed the results, contributed to the drafting of this manuscript, and approved the final manuscript.

## Conflict of Interest

The authors declare that the research was conducted in the absence of any commercial or financial relationships that could be construed as a potential conflict of interest.
